# High Pressure Steam

**Published:** 1880-12

**Authors:** 


### HIGH PRESSURE STEAM.

This question is being successfully demonstrated by the steamer Anthracite, where the steel boiler is constructed to stand fifteen hundred pounds pressure, but to run regularly with five hundred. This was done with coal at a saving, but petroleum is to be the fuel of the near future; the difficulty of controlling its intense heat has been found to be serious, as a petroleum flame impinging on iron soon causes it to weaken. Dr. Duryee has apparently overcome this in his blow-pipe principle, where he forces a mixed air and oil blast *through* a central flue of a long cylinder boiler. As the gases are mixed in a fire-brick furnace before entering the boiler, the danger of burning the iron is obviated.

We predict wonderful results from this, as a flame forty feet long through an eight-inch flue will afford about a hundred horse-power at a cost of five gallons of oil per hour.

